# Evaluation of radioactive ^125^I seed implantation for the treatment of refractory malignant tumours based on a CT-guided 3D template-assisted technique: efficacy and safety

**DOI:** 10.1186/s12885-020-07223-3

**Published:** 2020-08-03

**Authors:** Guang Sheng Zhao, Song Liu, Liang Yang, Chuang Li, Ruo Yu Wang, Jun Zhou, Yue Wei Zhang

**Affiliations:** 1 Tumor Center, Affiliated Zhongshan Hospital of Dalian University, No.6 Jie Fang Street, Dalian, 116001 Liaoning Province China; 2 Linyi Cancer Hospital, 6 East Lingyuan Street, Linyi, 276001 Shandong Province China; 3 Affiliated Zhongshan Hospital of Dalian University, No.6 Jie Fang Street, Dalian, 116001 Liaoning Province China; 4 Hepatobiliary and Pancreatic Center, Beijing Tsinghua Changgung Hospital, 168 Litang Road, Changping District Beijing, 102218 China

**Keywords:** ^125^I seed, Refractory malignant tumours, 3D template, Efficacy, Safety

## Abstract

**Background:**

To observe the medium- and long-term clinical efficacy and safety of radioactive ^125^I seed implantation for refractory malignant tumours based on CT-guided 3D template-assisted technique.

**Methods:**

Twenty-five patients with refractory malignant tumours who underwent radioactive ^125^I seed implantation based on CT-guided 3D template-assisted technique were selected. The post-operative adverse reactions were recorded. The number of puncture needles and particles used in the operation, dosimetric parameters, post-operative physical strength scores, and tumour response were statistically analysed. The overall survival time and survival rate were calculated, and the effect and prognosis were assessed.

**Results:**

^125^I seed implantation was successful in all patients without serious complications. The average number of implanted puncture needles was 17 (19.12 ± 13.00), and the median number of particles was 52 (55.12 ± 32.97). D_90_ in the post-operative clinical target volume (CTV) (93.24 ± 15.70 Gy) was slightly lower than that in the pre-operative CTV (93.92 ± 17.60 Gy; *P* > 0.05). The D_90_ in the post-operative planning target volume (PTV) (142.16 ± 22.25 Gy) was lower than the pre-operative PTV (145.32 ± 23.48 Gy; *P* > 0.05). The tumour responses at 6 months post-operatively: complete remission (CR), 20% (5/25); partial remission (PR), 48% (12/25); stable disease (SD), 24% (6/25); progressive disease (PD), 8% (2/25); CR + PR, 68% (17/25); and local control rate, 92% (23/25). The 6-, 12-, and 24-month survival rates were 100, 88, and 52%, respectively. The post-operative physical strength score (Karnofsky performance score, KPS) exhibited a gradual trend towards recovery, which rose to the highest value 12 months after implantation and then decreased slightly, but the average score was still > 90 points. There was one intra-operative pneumothorax, and two patients with superficial malignant tumours developed skin ulcerations. Multivariate analysis of prognosis showed that tumour sites and types were independent risk factors affecting survival. The number of needles and particles and template types were not the factors.

**Conclusions:**

3D template combined with CT-guided radioactive ^125^I seed implantation can improve the rational distribution of radiation dose in the tumour target area because accurate radioactive ^125^I particle implantation was achieved. This technique has fewer complications and can further extend the overall survival and improve the quality of life.

**Trial registration:**

Registration number: ChiCTR2000034566 2020/7/10 0:00:00

Retrospectively registered.

## Background

With the advent and clinical application of 3D printing technology, radioactive ^125^I seeds have improved the treatment of malignant tumours. ^125^I seed implantation assisted by a 3D template has made the treatment of malignant tumours more precise and has significantly reduced the complication rate. ^125^I seed implantation assisted by a 3D template has become an effective means for the treatment of advanced malignant tumours, especially in the treatment of refractory malignant tumours, including brain metastases, pancreatic cancer, and soft tissue tumours, as well as advanced tumours with post-operative recurrence and metastasis [1–4]. With technological advances, the types and volumes of malignant tumours treated by radioactive ^125^I seed implantation have gradually increased. It has not been reported, however, whether seed implantation for the treatment of malignant tumours increases the incidence of severe complications, such as implant and distant metastases, due to the increased use of transplant needles during surgery.

## Methods

### General clinical data

From August 2016 to March 2017, a total of 25 patients underwent seed implantation in our hospital, including 17 male and 8 female patients. The average age was 65 years (64.64 ± 14.12 years), and the age range was 44–87 years. Seven patients had lung tumours, 6 had bone metastases, 2 had pancreatic cancer, 1 had cervical lymph node metastases and 1 had inguinal lymph node metastases, 2 had bladder cancer recurrence, 1 had pelvic metastases, 1 had lung cancer with adrenal gland metastases, 1 had maxillary sarcoma, 1 had lung cancer with liver metastases, 1 had vulvar cancer recurrence, and 1 had liver cancer with brain metastases. The pre-operative physical strength score (Karnofsky performance score, KPS) was > 60, the white blood cells (WBC) count was ≥4.0 × 10^9^/L, and the expected survival time was > 3 months. Patients with tumour progression after radiotherapy and chemotherapy or patients who could not receive chemoradiotherapy were included. All patients were aware of their disease status and understood the possible treatment effect and adverse reactions. All patients voluntarily accepted the treatment method and signed a consent form for seed implantation surgery. The study was approved by the Ethics Committee of our hospital.

### Materials and devices

Domestic radioactive ^125^I seeds have a half-life of 60.2 d, an activity of 0.6–0.8 mCi (1 Ci = 3.7 × l0^10^ Bq), and a γ-ray energy of 27–35 keV were used. A brachytherapy treatment planning system (BTPS) (Beijing Astro Technology Ltd., Co., Beijing, China) was used, domestic particle puncture needles (Japan Baguang Company, Japan), a TRH-BXQ implant gun (China), a TRH-J implant positioning navigation device, and a GE 64-row spiral CT were used.

### Planning before BTPS surgery

The parameters (planned target dose (PTD), particle activity, and CT data) were inputted into the BTPS to simulate needle insertion and develop a pre-operative plan from which additional parameters were derived. The pre-operative plan was completed jointly by the operator and the physicist after a thorough discussion, and the patient imaging position was adjusted to the actual operating position to ensure that the intra-operative needle insertion was consistent with the pre-operative plan. The PTD was controlled to be 110–180 Gy, and the clinical target dose (CTD) was controlled at 80–100 Gy.

### ^125^I seed implantation technology method

The position of the patients was determined according to the position of the tumour. A CT scan was used for localization. Local and intravenous anaesthesia was administered. The positioning navigation device was installed according to the surface marking laser positioning line, the template was installed and adjusted, the position and angle of the needle insertion was controlled, and the needle channel was established according to the pre-operative plan. Beginning in the centre plane of the tumour, the needles were arranged in layers with a lateral margin of 1 cm and a depth of 0.5 cm from the distal edge. A CT scan was performed to determine the exact position, and the implantation was completed layer-by-layer with the implant gun. The particles were > 1 cm away from the skin to avoid damage to the skin. If necessary, an intra-operative planning correction and target dose optimization were performed.

### Post-operative dose verification

Additional parameters were input to the BTPS for particle reconstruction and post-operative dose verification. The relevant dosimetric parameters of the clinical target volume (CTV) and the planned target volume (PTV) 1 cm outside the CTV before and after surgery were calculated, including the dose of 90% of the target volume (D_90_), 90% of the prescribed dose (CTD and PTD) covering the target volume (V_90_), 100% of the prescribed dose (CTD and PTD) covering the target volume (V_100_), 150% of the prescribed dose (CTD and PTD) covering the target volume (V_150_), the conformal index (CI), and the external index of the target index (EI). Based on the American Association of Brachytherapy Association standards, the D_90_ should reach or exceed the PTD (V_100_ ≥ 90%); otherwise, the D_90_ was not satisfied. The dose parameters before and after particle implantation surgery are shown in Tables 1 and 2.

**Table 1:** Comparison of dosimetric parameters of clinical target volume (CTV) before and after seed implantation of 25 patients with refractory malignant tumor

Parameters	Before surgery			After surgery			P
	Interval	Median	Mean value	Interval	Median	Mean value	
D90	50–112	93	93.92 ± 17.60	54–116	106	93.24 ± 15.70	0.28
V100	42–90.8	74.3	71.68 ± 13.34	43.7–91	70.1	72.05 ± 11.70	0.26
V150	21.7–61.2	44.8	42.88 ± 11.40	23.9–62	43.1	43.09 ± 11.05	0.44
CI	0.4–0.82	0.63	0.63 ± 0.10	0.43–0.79	0.63	0.62 ± 0.08	0.14
EI	0.01–0.25	0.05	0.08 ± 0.07	0.01–0.31	0.07	0.08 ± 0.07	0.46
HI	0.3–0.64	0.4	0.42 ± 0.09	0.26–0.61	0.4	0.39 ± 0.08	0.41

**Table 2:** Comparison of dosimetric parameters of panned target volume (CTV) before and after seed implantation of 25 patients with refractory malignant tumor

Parameters	Before surgery			After surgery			P
	Interval	Median	Interval	Median	Interval	Median	
D90	114–181	148	145.32 ± 23.48	105–180	128	142.16 ± 22.25	0.39
V100	76.6–100	96.3	94.63 ± 5.76	73.7–100	95	93.47 ± 6.29	0.33
V150	46.2–90.4	64.5	67.35 ± 14.07	46.7–89.6	66.1	66.72 ± 12.11	0.23
CI	0.4–0.8	0.66	0.63 ± 0.11	0.42–0.82	0.61	0.63 ± 0.11	0.42
EI	0.18–1.1	0.42	0.46 ± 0.27	0.21–1.3	0.46	0.50 ± 0.29	0.38
HI	0.09–0.61	0.31	0.31 ± 0.13	0.1–0.59	0.34	0.31–0.12	0.40

### Post-operative treatment and observation

The post-operative physical score (KPS) and adverse reactions were recorded. The tumour response was followed for 6 months according to the Response Evaluation Criteria in Solid Tumors (version 1.1), including complete remission (CR: all target lesions disappeared.), partial remission (PR: The total length of diameter of the baseline lesions decreases > 30%), progressive disease (PD: The total length of diameter of the lesion increases > 20% or a new lesion appears.), stable disease (SD: The decreasing of lesions is not sufficient for PR, or the increasing of lesions is sufficient for PD.), effective rate (CR + PR), and local control rate (CR + PR + PD). The survival time and survival rates at 6, 12, and 24 months were noted and summarized, and the follow-up evaluations concluded in March 2019 or at the time of patient death.

#### Statistical analysis

Statistical analysis was performed using SPSS 20.0 software (International Business Machines Corporation, NewYork). Data are expressed as the mean ± standard deviation ($\overline{x}\pm s$), M (median), L (Lower limit) ~ U (Upper limit) or percentage. Paired t test was used for preoperative planning and postoperative verification of dosimetric parameters. The KPS performance scores at different time points before and after treatment were analyzed by repeated measures analysis of variance. Kaplan-Meier survival analysis was used to evaluate OS. OS was calculated from the day that their ^125^I was started until their reported death date. For analysis of OS, patients who were known to have been alive at the end of the study period were censored at this endpoint (March 31st, 2019). Related factors were analyzed using single factor and multi-factor Cox risk regression models. *P* < 0.05 was considered statistically significant.

## Results

### Comparison of pre- and post-operative dosimetric parameters

**Fig. 1:**
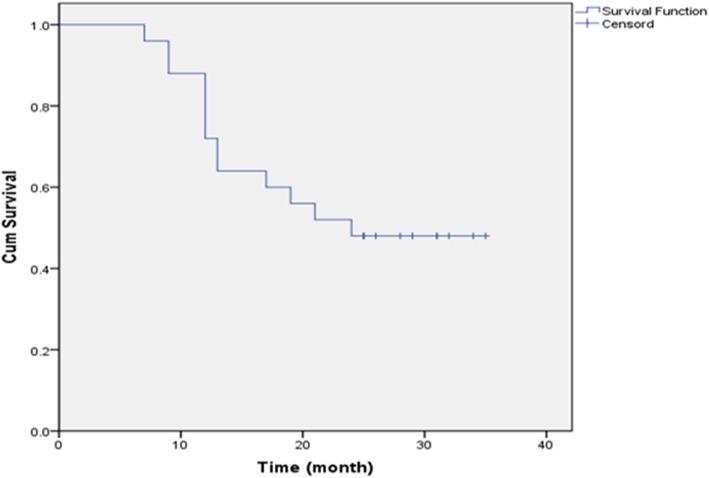
Surgical procedure of ^125^I seed implantation for lung cancer and follow-up imaging. A: Image of eldrly patient with lung adenocarcinoma, lung tumor invading the rib, CT localization and target area delineation image before seed implantation; B: Template assisted CT-guided needle puncture, it can be observed that the needle angle and the depth is good; C: After seed implantation, CT image showed good particle distribution in the lesion, and the dose distribution was basically consistent with the preoperative plan; D: Reviewed at 3 days after intervention, CT showed uniform particle distribution in the lesion area; E: 6 months after seed implantation, CT showed that the lesions were significantly reduced to complete remission, a small number of residual lesions could be observed; F: 2 years after intervention, CT showed complete disappearance of lesions, and localized aggregation of implanted particles; G: Planed verification charts of dose before seed implantation; H: Verification charts of dose after seed implantation, and the dose parameters are basically the same with those before implantation

Under the guidance of a 3D template-assisted CT, seed implantation was successfully completed in all patients, and the implantation process was uncomplicated. The average number of puncture needles implanted was 17 (19.12 ± 13.00), and the median number of particles implanted was 52 (55.12 ± 32.97). The D_90_ of the post-operative CTV was 93.24 ± 15.70 Gy, which was slightly lower than that of the pre-operative CTV (93.92 ± 17.60 Gy), but there was no significant difference between the two groups (*P* > 0.05). The D_90_ of the post-operative PTV was 142.16 ± 22.25 Gy, which was lower than that of the pre-operative PTV (145.32 ± 23.48 Gy), but there was no significant difference between the two groups (*P* > 0.05). The pre- and post-operative CTV dose parameter, EI, was close to zero. There were no significant differences in other related dosimetric parameters (*P* > 0.05, Tables 1 and 2), and the post-operative verification results were considered satisfactory. Figure 1 shows the surgical procedure for ^125^I seed implantation in patients with lung cancer and the follow-up imaging.

## Tumour responses after seed implantation

CT or MRI was conducted 1, 2, 4, and 6 months after surgery for dynamic imaging observations. Tumour response was evaluated 6 months after surgery in combination with radioactive ^125^I seed attenuation characteristics. The tumour responses 6 months post-operatively were as follows, as shown in Table 3: CR, 20% (5/25); PR, 48% (12/25); SD, 24% (6/25); PD, 8% (2/25); effective rate (CR + PR), 68% (17/25); and local control rate, 92% (23/25).

**Table 3:** Evaluation of tumor response in patients at 6 months after seed implantation

CR		PR		SD		PD		Objective remission		Disease control	
n	%	n	%	n	%	n	%	n	%	n	%
5	20	12	48	6	24	2	8	17	68	23	92

*CR* complete remission, *PR* partial remission, *SD* stable disease, *PD* progressive disease

### Statistics on the survival time of patients

**Fig. 2:**
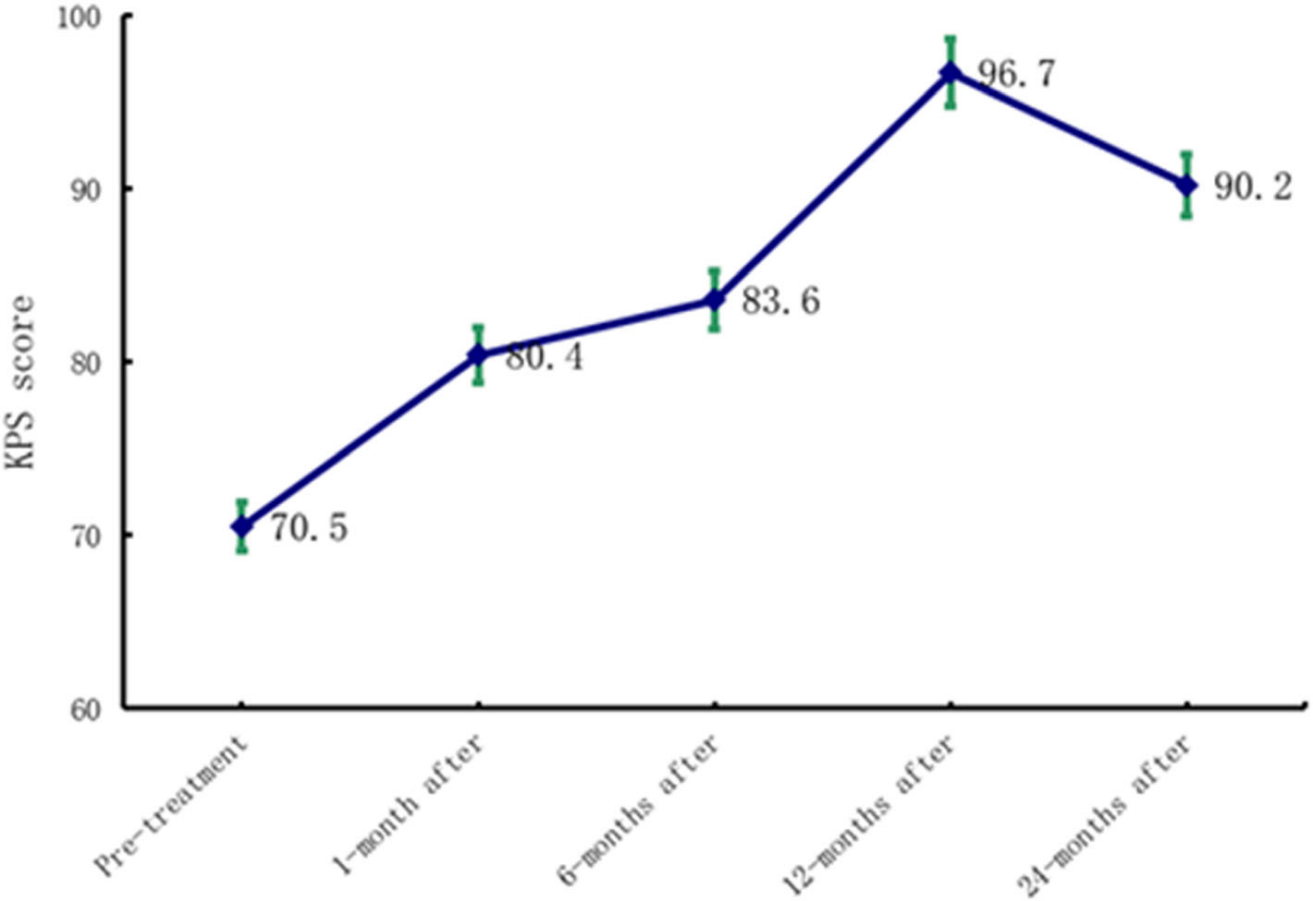
Median survival time of the entire group of patients

None of the patients in either group were lost to follow-up. All patients were followed according to the plan, and the follow-up evaluation data were concluded in March 2019. The 6-, 12-, and 24-month survival rates were 100% (25/25), 88% (22/25), and 52% (13/25), respectively (Table 4). The median survival time for the entire group of patients was 24 months (Fig. 2).

**Table 4:** The survival time and survival rate of patients after seed implantation

6-month survival rate		12-month survival rate		24-month survival rate		Median survival time
n	%	n	%	n	%	24.00
25	100	22	88	13	52

### Physical strength score (KPS) and adverse reactions

**Fig. 3:**
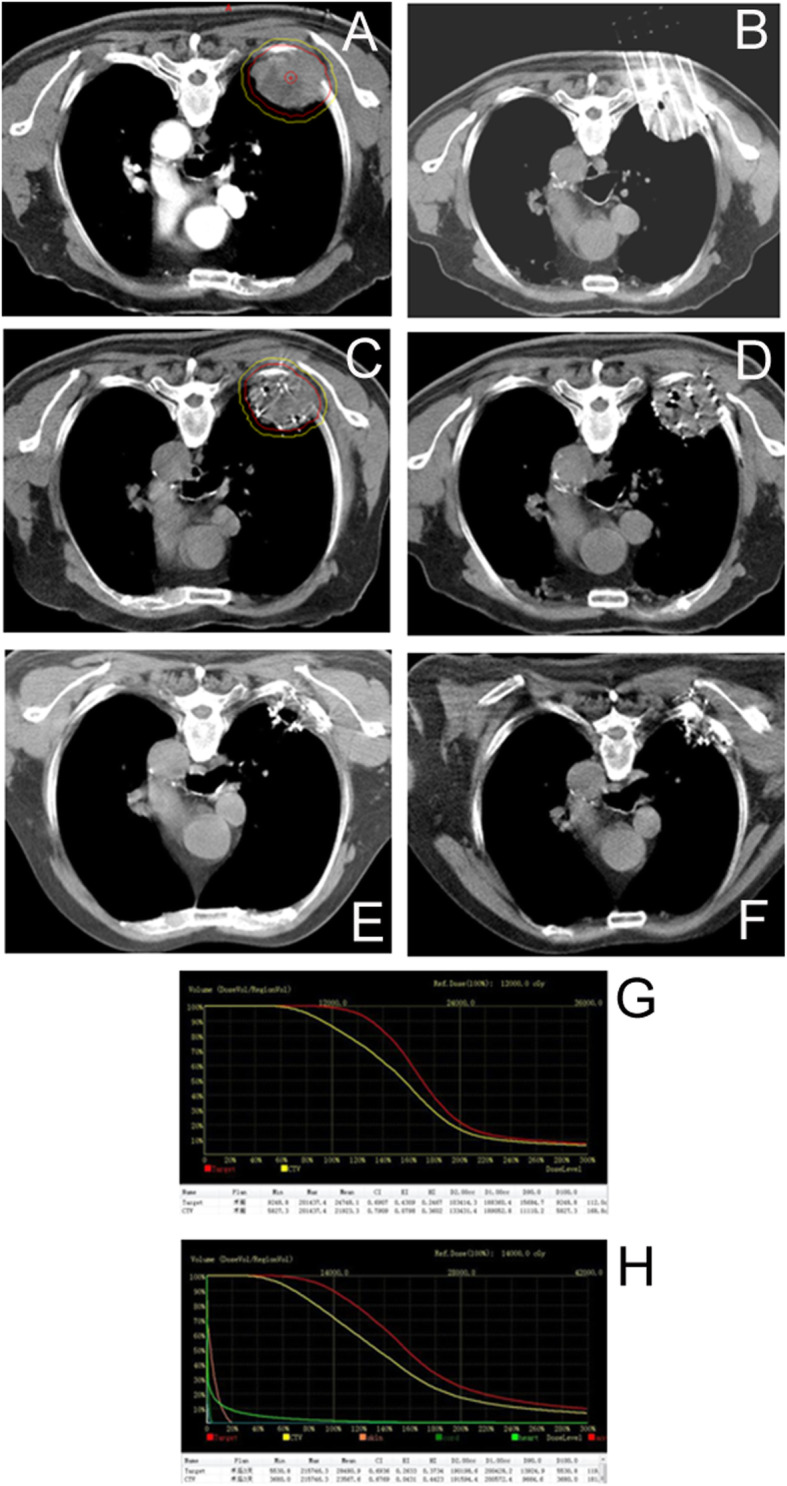
KPS score

The physical strength score (KPS) of the entire group gradually recovered and increased, reached the highest value 12 months after seed implantation, and then decreased slightly; however, the mean KPS score was still > 90 points (Fig. 3). There was a significant difference between the two groups (*F* = 6.428, *P* = 0.003 < 0.05). One patient with CR had an intra-operative pneumothorax that was treated with closed pleural drainage. Two patients with superficial malignant tumours and skin ulcerations were treated symptomatically; the scars healed by 6 months post-operatively. There were no uncontrollable major haemorrhages in the entire group and no serious complications, such as puncture or implant metastases post-operatively.

### Prognostic multivariate analysis

The log-rank test was used for univariate analysis, and the Cox model was used for multivariate analysis. Univariate and multivariate analyses included age, sex, template type, number of puncture needles, number of particles, and tumour location and type for the 25 patients. The location and type of tumour were independent risk factors for median overall survival (mOS), but the number of puncture needles and particles were not factors that affected the prognosis of patients (Table 5).

**Table 5:** Multivariate analysis on the factors that affect the treatment prognosis

Factors	n	Univariate			Multivariate		
		HR	95%CL	P	HR	95%CL	P
Age							
< 60 years old	9	1			1		
≥ 60 years old	16	0.985	0.546–1.809	0.961	0.853	0.460–1.581	0.621
Gender							
Male	17	1			1		
Female	8	0.746	0.390–1.425	0.375	0.934	0.457–1.740	0.813
Template type							
Coplanar	22						
Non-coplanar	3	0.823	0.460–1.581	0.623	0.924	0.461–1.811	0.816
Number of puncture needle							
< 10	6	1			1		
≥ 10 < 20	9	1.614	0.849–3.068	0.144	1.784	0.673–4.729	0.244
≥ 20	10	1.001	0.484–2.070	0.998	0.669	0.256–1.746	0.411
Number of seed implantation							
< 30	7	1			1		
≥ 30 < 60	9	1.241	0.765–2.012	0.381	1.024	0.719–1.459	0.896
≥ 60	9	1.567	1.095–2.240	0.067	0.633	0.357–0.892	0.402
Tumor location							
Thoracic tumor	7	1			1		
Bone and soft tissue tumor	7	0.423	0.219–0.816	0.012	0.147	0.042–0.527	0.003
Abdominal tumor	4	0.167	0.051–0.634	0.008	4.995	2.557–9.582	0.003
Genitourinary tumor	4	0.342	0.192–1.357	0.019	0.297	0.119–0.731	0.007
Lymph node metastasis tumor	2	0.641	0.049–0.829	0.012	0.627	0.047–0.359	0.009
Head and neck cancer	1	0.378	0.161–0.653	0.026	0.254	0.103–0.545	0.001

Values are the risk ratio (95% confidence interval) of the generalized linear model, which reflects that tumor location is the factors affecting prognosis. Model is adjusted for age, gender, number of puncture needle, number of seed implantation, tumor-location. The *p*-values were defined as< 0.05

## Discussion

The incidence of malignant tumours has increased year after year worldwide [5, 6]. In 2008, US President Barack Obama introduced the concept of precision medicine. In the following 10 years, the concept of precision medicine has been shown to have enormous value and has given oncologists more hope and choices. Nevertheless, there are a large number of immunosuppressive substances or factors in the tumour microenvironment that impair the immune system from functioning normally. At present, the precise radical treatment of malignant tumours at the genetic level cannot be achieved. As the product of several minimally invasive disciplines, radioactive ^125^I particle implantation technology is a relatively accurate treatment in clinical practice. Radioactive ^125^I particle implantation technology has developed rapidly in recent years, the application range of which covers nearly all types of malignant solid tumours, including common brain metastases, lung cancer, pancreatic cancer, liver cancer, bone metastases, and various metastatic lymph node and soft tissue tumours [1–4, 7–12]. Due to the need to adjust the needle during surgery, the larger the tumour is, the greater the number of implants needed. Indeed, there are no domestic reports that have determined whether puncture needles promote the release and escape of tumour cells, thus leading to complications, such as puncture tract transfer and distant organ metastases.

In the current study, 25 patients with advanced refractory malignant tumours underwent continuous seed implantation, and no serious complications occurred in the entire group, such as implant and distant organ metastases. Thus, although the number of implanted needles was increased, the application of 3D printing technology rendered template-assisted seed implantation accurate, shortened the operative time, and decreased the number of intra-operative needle adjustments; as a result, the complications caused by repeated punctures were decreased. The study further illustrated the feasibility and safety of radioactive ^125^I seed implantation with 3D template guidance for the treatment of malignant tumours.

With the pre-operative use of the BTPS to develop a rational treatment plan and the implementation of the treatment plan intra-operatively, CT-guided 3D template-assisted ^125^I seed implantation for the treatment of malignant tumours is more accurate [4, 13–15] and extends the survival time and quality of life of patients with advanced malignancies. Mo et al. [11] applied CT-guided seed implantation combined with chemotherapy to treat metastatic soft tissue tumours after 4–6 cycles of first-line chemotherapy. The results showed that the 1- and 2-year survival rates were 46.7 and 28.9%, respectively, while the 1- and 2-year survival rates of the control group with second-line chemotherapy were 6.3 and 0% [11]. Although the overall survival time was 16.9 ± 5.01 and 12.1 ± 4.8 months for the two groups and there was no significant difference between the groups, the experimental group had a significantly improved symptom remission rate and quality of life [11].

Wang et al. [12] utilized ^125^I seed implantation in the treatment of pelvic metastases and showed that the 1- and 2-year survival rates were 81.8 and 45.5%, respectively. The results of Wang et al. [12] were consistent with the results reported herein. All 25 patients in this study had refractory advanced malignant tumours that progressed after radiotherapy or chemotherapy or were unable to undergo radiotherapy and chemotherapy. The 1- and 2-year survival rates were 88 and 52%, respectively, and the median survival time was 24 months, which were higher than the results reported by other similar studies. The local control rate of the tumour was 92% 6 months after surgery. This result is difficult to achieve in patients with advanced refractory malignant tumours; the result was demonstrated by a gradual increase in the physical strength score (KPS). No patients were administered systemic chemotherapy or other treatments from seed implantation to the completion of follow-up. The analysis of prognostic factors in this study also suggested that the tumour site and type are influential factors for CT-guided 3D template-assisted ^125^I particle implantation technology, and other factors, such as template type, are not factors that affect prognosis [13].

With the development and clinical application of gene sequencing technology, the treatment of malignant tumours is more comprehensive and precise, which further improves the clinical benefit of patients with malignant tumours [16, 17]; however, the multidisciplinary treatment model is still preferred for the treatment of malignant tumours. A single method often has less of an effect in the treatment of tumours. Patients with refractory advanced malignancies, including patients with progression after chemoradiotherapy and patients who are not suitable for chemoradiotherapy and end-stage chemotherapy, have a very poor prognosis. The expected survival time of such patients is approximately 3 months [18, 19]. Radioactive ^125^I seed implantation is a more accurate radiotherapy technique for the treatment of malignant tumours. Radioactive ^125^I seed implantation is guided by imaging to implant radioactive ^125^I seeds into the tumour through a puncture needle so that the particles disseminate radioactivity inside the tumour. This method has a long-lasting effect, and the side effects of this method are significantly lower than those of other radiotherapy methods. With the clinical development and application of 3D printing technology, CT-guided 3D template-assisted ^125^I seed implantation technology further improves the efficacy of radiation in tumour target areas while sparing surrounding vulnerable tissues and organs. Needle puncture and arrangement rely entirely on surgeon experience. Due to an inability to effectively control quality, it is relatively easy to have a localized cold dose of the tumour, which inevitably leads to tumour progression.

The results of this study showed that the D_90_ of the PTV and CTV target areas were not significantly different from the corresponding pre-operative values, which further indicated that the method can improve the actual dose distribution. At the same time, the V_100_ and V_150_ parameters were not significantly different from the pre-operative plan. Considering that seed implantation is under template control, bleeding and motion artefacts are decreased; thus, the target dose is precisely controlled on the basis of better control of the tumour target volume, which is consistent with recent reports in the literature [13, 14].

The data also suggest that 3D printed coplanar and non-coplanar templates and the number of needles and particles do not influence prognosis, further suggesting the safety of this treatment for advanced malignant tumours. Therefore, as a precise comprehensive treatment, a 3D template combined with CT-guided radioactive ^125^I seed implantation can be repeated for the treatment of recurrent tumours [20]. Moreover, we also observed that the EI value of the CTV target correlation index was close to 0, suggesting that optimizing the clinical target area may be more valuable in reducing peripheral tissue damage and increasing the actual intratumour particle dose distribution. This finding may also be the main reason for the good efficacy demonstrated in the current study. The importance of this observation and dose study of the CTV target area has not been previously reported.

CT-guided ^125^I seed implantation in the treatment of malignant tumours is included in the treatment protocols in China, which makes this treatment more standardized [21]. In the current study, the long-term efficacy and safety of the CT-guided 3D template-assisted ^125^I seed implantation technique in the treatment of malignant tumours for refractory malignancies was confirmed, and a 2-year clinical follow-up observation combined with post-operative verification of relevant dosimetric parameters further confirmed the clinical efficacy and safety of the technique as a rational form of treatment. Changes in the number of circulating tumour cells in peripheral blood tumours post-operatively were not determined in the current study [22]. Whether this method can promote tumour micrometastasis is still uncertain, and the number of samples in this study was small, which was also a shortcoming of this study.

## Conclusions

In conclusion, this preliminary study showed the safety and efficacy of CT-guided 3D template-assisted ^125^I seed implantation in the treatment of malignant tumours, the rationality of radiologic dose division, and the delivery of a safe and effective treatment to patients with refractory advanced malignant tumours, which is worthy of further clinical application.

## Data Availability

The datasets used and/or analysed during the current study are available from the corresponding author on reasonable request.

## Abbreviations

CTV: Clinical target volume
PTV: Planning target volume
CR: Complete remission
PR: Partial remission
SD: Stable disease
PD: Progressive disease
KPS: Karnofsky performance score
PTD: Planned target dose
CTD: Clinical target dose
CI: Conformal index
EI: Target index
mOS: Median overall survival
WBC: White blood cells
